# Stakeholder-engaged research: strategies for the prevention and control of overweight and obesity in Kenya

**DOI:** 10.1186/s12889-021-11649-0

**Published:** 2021-09-06

**Authors:** Mary Njeri Wanjau, Lucy W. Kivuti-Bitok, Leopold N. Aminde, J. Lennert Veerman

**Affiliations:** 1 University of Nairobi, School of Nursing Sciences, Nairobi, Kenya; 2 Griffith University, School of Medicine, Gold Coast, Queensland Australia; 3 Non-communicable Disease Unit, Clinical Research Education, Networking & Consultancy, Douala, Cameroon

**Keywords:** Stakeholder-engagement, Prevention, High body mass, Overweight, Obesity, Indigenous foods, Kenya, Low- and middle-income

## Abstract

**Background:**

This study was done as part of a larger study that aims to identify the most impactful and cost-effective strategies for the prevention and control of overweight and obesity in Kenya. Our objective was to involve stakeholders in the identification of the strategies that would be included in our larger study. The results from the stakeholder engagement are analyzed and reported in this paper.

**Design:**

This was a qualitative study. A one-day stakeholder workshop that followed a deliberative dialogue process was conducted.

**Participants:**

A sample of stakeholders who participate in the national level policymaking process for health in Kenya.

**Outcome measure:**

Strategies for the prevention and control of overweight and obesity in Kenya.

**Results:**

Out of the twenty-three stakeholders who confirmed attendance, fifteen participants attended the one-day workshop. The stakeholders identified a total of 24 strategies for the prevention and control of overweight and obesity in Kenya. From the ranking process carried out the top six strategies identified were: a research-based strategy for the identification of the nutritional value of indigenous foods, implementation of health promotion strategies that focus on the creation of healthy environments, physical activity behavior such as gym attendance, jogging, walking, and running at the individual level, implementation of school curricula on nutrition and health promotion, integration of physical education into the new Competency-Based Education policy, and policies that increase use of public transport.

**Conclusion:**

The stakeholders identified and ranked strategies for the prevention and control of overweight and obesity in Kenya. This informs future overweight and obesity prevention research and policy in Kenya and similar settings.

**Supplementary Information:**

The online version contains supplementary material available at 10.1186/s12889-021-11649-0.

## Background

Globally, high body mass index (BMI) accounted for 4.0 million (2.7–5.3) deaths and 120 million (84–158) disability-adjusted life years (DALYs) among adults [1]. In sub-Saharan Africa, it is predicted that prevalence of overweight and obesity will increase over the next few decades at a faster pace than elsewhere in the world [2]. The latest published cross-sectional household survey in Kenya indicates that 27% of the adult population is overweight or obese (38.5% women and 17.5% men) [3]. Notably, the global burden of disease study ranks high body mass among the top 10 risk factors that contributed to total DALYs in Kenya. As a leading risk factor for disease in Kenya, high body mass registered the highest percentage increase of 67.3% between 1997 and 2017, from 486 DALYs per 100,000 to 812 DALYs per 100,000 [4]. Combined with the persisting burden of infectious diseases such as tuberculosis, malaria, and HIV/AIDS, the increasing prevalence of high body mass and related non-communicable diseases (NCDs) has created a double burden of disease within a strained health system.

Strategies that target the modifiable components of energy intake (diet) and energy expenditure (physical activity) result in favorable BMI trends [5, 6]. In Kenya, current prevention and control strategies for overweight and obesity focus on both the individual and environmental factors that predispose one to high body mass [7]. Since there are multiple potentially effective strategies it is important to ensure that resource allocation choices lead to optimal health for the available budget. Policymakers in Kenya are keen to make rational, transparent, evidence-based health choices for the control and prevention of high body mass. In the past, choices have often been driven by political, historical, or commercial imperatives, but the importance of ‘evidence-based policy’ is increasingly being recognized [8]. In addition, various global strategies have been proposed to guide action on the prevention and control of overweight and obesity [9]. However, there is a need for the context-specific evaluation of the effectiveness, feasibility of widespread implementation, and sustainability of these strategies [1, 6].

Against this background, we designed a study that applies modelling techniques to evaluate a selected number of strategies to generate research-based evidence on the most impactful and cost-effective strategies for the prevention and control of overweight and obesity in Kenya. We applied the assessing cost-effectiveness (ACE) approach to priority setting, which defines areas of action where the greatest health gains can be achieved for available resources [10, 11]. Stakeholder engagement is part of the due process in the ACE approach. The engagement process allows for the incorporation of multiple perspectives in the planning and execution of studies [12]. Input from other people beyond the traditional research team often improves the quality of research. Also, it brings in transparency throughout a research project and assists in the promulgation of the findings, accelerating their adoption into practice [10, 13]. Concannon and colleagues [13] refer to this as the process of moving research evidence off bookshelves and into practice. The stakeholder workshop reported in this paper was part of this larger ACE stakeholder- engaged study. Specifically, for this stage of engagement, we aimed to involve stakeholders in the identification of strategies for the prevention of overweight and obesity in Kenya that would be modelled in our ACE study. In this context, a stakeholder was described as “an individual or group that is responsible for or affected by health- and healthcare-related decisions that can be informed by research evidence” [13].

Our paper also seeks to contribute to the evidence surrounding stakeholder-engaged research in Public Health.

## Methods

### Study design

This was a qualitative study that followed a workshop approach. We conducted a one-day stakeholder workshop that followed a deliberative dialogue process [14]. We considered this the most appropriate approach because our investigation was exploratory [15].

### Conceptual framework

Though the standards for reporting qualitative research (SRQR) reporting guideline guided the writing of this paper [16], we used the Ray and Miller framework [12] for reporting our stakeholder engagement process. This framework provides great rigor, transparency, and consistency in the reporting of stakeholder-engaged research. The Ray and Miller’s framework covers three main topics: contexts, processes and outcomes. Under outcomes, the immediate, intermediate, and long-term outcomes are reported. Since the study is still ongoing, we only report the intermediate outcomes that we have achieved so far. We report on planned and actual processes.

### Context

This study was planned in the context of an overall research agenda to identify the most impactful and cost-effective strategies for the prevention and control of overweight and obesity in Kenya. In this engagement, we solicited the stakeholders’ interests, views, values, knowledge, and experiences. This desired input from stakeholders was informed by best practices of stakeholder engagement [13]. Specifically, we asked the stakeholders to 1) in groups, identify existing or new strategies that they would propose for the prevention and control of high body mass in Kenya; and 2) as individuals, identify the top three strategies that they would propose for inclusion in our ACE modelling study and rank them from number 1 to 3, with 1 being the strategy with the highest priority. We considered the engagement as a bi-directional relationship between ourselves (researchers) and stakeholders. The results of this phase would inform the selection of strategies that would be included in our ACE study.

### Processes

#### Stakeholder recruitment and composition

Since the larger ACE study seeks to inform the prioritization of strategies for the prevention and control of overweight and obesity in Kenya, we opted to have our stakeholders as individuals who are known to participate in policymaking at the national level. This is consistent with the established description of stakeholders [13]. In our recruitment, we applied purposive and snowball sampling techniques. To capture a variety of perspectives we targeted a multisectoral representation of decision-makers. We considered that the public would be effectively represented by stakeholders from civil society organizations. Table 1 gives a description of potential stakeholders that guided the recruitment process.

**Table 1:** Description of potential stakeholders

Overall description	
Members of teams that look at preventive and early intervention strategies for NCD control focusing on diseases or risk factors such as high body mass.	
Description of various stakeholders	
• Head of Division NCDs, Ministry of Health (MoH), Kenya	
• Head of Health Promotion Unit, MoH, Kenya	
• Standards and Quality Assurance directorate, MoH	
• A member (or members) from any health advisory committees recommended by the MoH Heads of divisions above.	
• Other MoH officials - representatives from various divisions who would be involved in making choices of what strategies to implement and in what order. For example, officers from health economics, data, and statistics.	
• Representatives from other relevant agencies such as Kenya Medical Research Institute (KEMRI)	
• Representatives from influential and credible bodies that the MoH would recommend	
• Representatives from Civil Society	
• Medical Research Council representative	
• An officer from the treasury who interacts with the health budget or activities	
• A health counterpart in the Ministry of Planning, Ministry of Education, Science and Technology	
• External partners for example, WHO health representative overseeing NCD control or health promotion	
• Academic experts in health systems management and health economics drawn from universities in Kenya.	

We shared the description of stakeholders with two identified stakeholders, one from the MoH, and another from the University of Nairobi, Kenya. With the assistance of these two stakeholders, potential participants that fit the descriptions given were identified by name, and their official contact details supplied to us. The MoH stakeholder supplied us with a list of main policymakers, development assistance partners, and other key individuals involved in NCD control in Kenya. We also conducted online searches for information on persons in roles that fit our stakeholder description and acquired their email addresses through official ministry and organization websites. For some stakeholders, we contacted their colleagues in the various institutions to help us get in touch with them. Email communication was sent out to all identified individuals explaining to them what the purpose of the study was, requesting their participation, giving details of their role in the study, and emphasizing the voluntary and confidential nature of participation. The components of the email communication material were part of the ethics review and approval for this study. For the identified stakeholders who did not respond to the initial email communication, follow up was done through phone calls and emails. We were able to reach every identified stakeholder. A snowball method ensued with assistance from stakeholders from two leading civil society organizations involved in decisions for health in Kenya. In total, thirty-six initial invite emails were sent out. Thirty-five stakeholders confirmed their willingness to participate in the study. One stakeholder gave a tentative confirmation citing a busy schedule as the main hindrance for participation. A follow up email communication and an e-flier were sent to the thirty-six stakeholders inviting them to the stakeholder engagement process set to take place through a one-day workshop. The stakeholders were invited without prior knowledge of their specific views on the study topic. A total of twenty-three of the invited stakeholders confirmed their attendance. This surpassed our target of a minimum number of 13 stakeholders. We had aimed to have at least 13 stakeholders attending the workshop. This target was largely guided by the description of stakeholders (see Table 1).

#### Frequency and duration of engagement

A total of five planning meetings were held in Kenya before the workshop date. These were in-depth briefing sessions held to discuss the engagement process, workshop moderation, planning, and logistics. The meetings were held between the field researcher (MNW) and five stakeholders who represented the university sector, ministry of health, humanitarian aid sector, and the civil society. The university and ministry of health stakeholders were purposively selected as they were considered resourceful in the workshop moderation and planning process. The other three stakeholders were the first responders who had confirmed their willingness to participate in the study as stakeholders. The duration of the meetings varied guided by the purpose of each session that was held. On average, each meeting lasted for one and a half hours. No joint meetings were held before the workshop due to time and financial constraints. MNW took notes and kept a record of the deliberations and actions agreed upon. For purposes of planning and coordination, there were multiple telephone and email communications between the field researcher (MNW) and senior researcher (JLV), and between the field researcher (MNW) and the selected five stakeholders.

Two meetings were held after the workshop. One was a one-hour joint review meeting that the field researcher (MNW) held with four stakeholders immediately after the workshop. The same stakeholders engaged in the planning meetings before the workshop attended the first meeting after the workshop except for one stakeholder from the MoH, who had given an apology on the day and was not present at the workshop. The objectives of this meeting were: 1) to provide an opportunity to receive immediate feedback on the workshop that had taken place, 2) to discuss the feedback and document recommendations for areas that required improvement.

The second was a three-hour post-workshop meeting which was held between the field researcher and one stakeholder from the university sector. This was held several days after the workshop. The objective of the 2nd meeting held after the workshop was to develop a plan for the workshop report writing and subsequent publication of the workshop deliberations. Of the four stakeholders above, two stakeholders were invited to this meeting representing the university sector and the humanitarian aid sector. The stakeholder from the humanitarian aid sector passed an apology on the day. The stakeholder present from the university sector was considered adequate to achieve the meeting’s objective. In both meetings, guided by the meeting objectives, the stakeholders and field researcher held open discussions and reached consensus on the various matters discussed. We present the outcomes of these meetings under our immediate outcomes section.

The engagement of stakeholders took place during a one-day workshop in October 2019. The workshop was conducted in English. On arrival, the participants filled out a registration form, read through the informed consent form and each signed a copy. For this session, the stakeholders were divided into two sub-groups with an average of seven members per sub-group. Each sub-group seated at one round table. The group sitting was informed by arrival time. As stakeholders arrived, they were guided to occupy the tables proximal to the podium. Initially a total of 3 tables were occupied. To achieve a balance in number of stakeholders per group, members in the 3rd table were distributed to the first and second tables. In sub-groups, the participants were asked to identify existing and new strategies for the prevention and control of overweight and obesity in Kenya.

From each sub-group, the stakeholders appointed someone to moderate the discussion, and another person was appointed to record the sub-groups discussion points on a flip chart. Each sub-group then presented their discussions to the broader group eliciting more dialogue from the broader group with additional new ideas and views emerging. For quality purposes, this larger discussion session was facilitated by the field researcher. A stakeholder had been assigned this role and been taken through an in-depth briefing before the workshop day. However, on the day, he sent an apology due to an urgent work commitment. In the presentations and deliberations, the participants were given the right to withdraw or add any identified strategies. Once the two subgroups had presented their discussions, we considered this our level of saturation for that workshop activity. We then carried out the final workshop activity that involved the ranking of the selected strategies. All the identified strategies were listed and displayed at the front of the room. We present the displayed lists as supplementary file 1 to this publication. Each stakeholder present was asked to identify the top three strategies that they propose for inclusion in our ACE modelling study and rank them from number 1 to 3, with 1 being the strategy with the highest priority. Each participant was given 3 colored stickers; gold, green, and blue. Each was asked to stick the golden sticker against the strategy that one ranked as number 1 (highest ranking). The green stickers against the strategy that one considered as number 2 and the blue sticker for the strategy that one ranked as number 3 strategy. The entire session lasted for about 3 h. To complement the flip chart recording, the workshop assistant and the field researcher took down notes. Additionally, with consent from the participants, the presentations to the larger group were audio-recorded.

### Data management

The workshop discussions were transcribed verbatim. The initial transcription was done by a 3rd party. Two authors (MNW, LKB) verified the validity of the transcription by listening to the audio recordings and comparing them with the transcripts. One author (MNW) did the necessary updates and corrections to the transcripts. This was checked by another author (LKB). The trustworthiness of our findings was enhanced by reading the transcriptions, flip chart recordings, and workshop notes multiple times. We generated a list of the strategies that were identified by stakeholders and noted any accompanying remarks made by stakeholders in the discussions that took place during the presentations to the larger group. As a research team, we applied reverse coding to put a weighting on the ranking done. We assigned a weighted score of 3 to any strategy that was ranked one (gold sticker) and strategies ranked second (green sticker) was assigned a weighted score of 2 and those ranked third (blue sticker) were assigned a weighted score of 1. A tally was done, and the total score put against each strategy. We then ranked the strategies from highest to lowest.

This stakeholder engagement process was approved by the Griffith University Human Research Ethics Committee (GU Ref No: 2019/707). All methods were performed in accordance with the relevant guidelines and regulations in the Griffith University Research Ethics Manual. The stakeholders completed a consent form before the workshop began.

## Results

### Processes

#### Stakeholder recruitment and composition

Out of the twenty-three stakeholders who confirmed attendance of the one-day workshop, fifteen (65%) participants were present. Table 2 presents the names of the organizations represented by the thirty-five stakeholders who confirmed their willingness to participate in the study. We indicate those who were present at the workshop and those who were absent with an apology.

**Table 2:** Summary of stakeholders engaged for the study by institution representation

Representation of stakeholders who attended the workshop	Additional institution representation of stakeholders who confirmed their willingness to participate in the study but were absent from the workshop
1. National Commission for Science, Technology, and Innovation 2. School of Nursing, University of Nairobi, Kenya 3. Ministry of Health (MoH), Kenya- Immunisation Department 4. MoH - Health Systems Department 5. Kenya Red Cross 6. The Non-Communicable Diseases Alliance Kenya 7. Institute of Diplomacy and International Studies, University of Nairobi, Kenya 8. Kenya National Commission on Human Rights 9. Strathmore University, Kenya 10. Kiambu County Health, Kenya 11. Dental School, University of Nairobi, Kenya 12. Mater Hospital, Kenya 13. Personal consultant in Public Health- supply chain management 14. Kenyan Network of Cancer Organizations 15. Oncology Nursing Chapter -Kenya	1. Standards and Quality Assurance directorate, MoH, Kenya 2. Tobacco Control Division, MoH, Kenya 3. NCDs Division, MoH, Kenya 4. Universal Health Coverage, Presidential Advisory & Strategy Unit. 5. Executive Office of the President 6. WHO – NCDs Unit in Kenya 7. PharmAccess Kenya 8. Personal Consultant- Psychologist 9. Swedish Workplace Programme, SWP 10. A former head of Preventive and Promotive Health Services, MoH

### Immediate outcomes

#### Outcomes from the planning meetings held before the workshop

In the meetings that took place before the workshop, several recommendations were made and incorporated in planning for the workshop. We present a list of these recommendations and actions taken in Table 3.

**Table 3:** Recommendations during planning meetings and action taken

Deliberations and recommendations made	Responses and action taken
Review of the flow and duration of activities in the workshop	Final program agreed upon (Supplementary file 2)
Discussion on participatory approach for the workshop moderation roles	Two stakeholders identified to facilitate discussions sessions
Proposal to complement documentation of workshop proceedings	Incorporated the audio recording of the workshop discussions. This was not in the original research plan.
Two venues were under consideration for the workshop	Venue was selected and agreed upon by all.
In-depth briefing on duties and roles for the workshop day	Selected stakeholders prepared for the allocated roles in the workshop. Details of this given under the methods section

#### Outcomes from the stakeholder workshop

In this section, we present the various prevention and control strategies that the stakeholders identified as relevant and appropriate for the prevention and control of overweight and obesity in Kenya. Where stakeholders gave additional comments and remarks regarding the identified strategies, we also report those remarks. Remarks captured included their thoughts on the effectiveness of current strategies, comments regarding their appropriateness, relevance, and feasibility. While the stakeholders expressed confidence in the effectiveness of some existing strategies, there was a fair amount of uncertainty expressed for many of the existing strategies. Guided by the Swinburn, Gill, & Kumanyika [17] framework that categorizes obesity determinants and solutions, the stakeholders discussed the level of intervention for the strategies proposed for the Kenyan setting. From the final list agreed upon by all stakeholders, each stakeholder present identified the top three strategies that they proposed for inclusion in our ACE modelling study. The total weighted scores guided the ranking process where strategies with higher weighting ranking top and those with lesser weighting appearing lower in the ranking. Table 4 presents a summary of the results.

**Table 4:** Identification and ranking of strategies for the prevention and control of overweight and obesity in Kenya

Strategy	Stakeholders’ comments/ remarks	Ranking process	
		Stickers awarded^a^	Total weighting^**b**^
1. Promotion of indigenous foods in Kenya Agricultural & Livestock Research Organisation (KALRO) [18]	Stakeholders proposed to have KALRO promote and coordinate research to identify the nutritional value of indigenous foods. The research-based evidence would then be used to promote indigenous foods found to have high nutritional value. This was considered a potential policy strategy as the research findings would guide policy in the agriculture and food industry. They considered that it would also potentially influence behavior patterns through the resulting health promotion and social marketing programs.	4 gold, 2 green	16
2. Health promotion and education to extend to all levels (beyond behaviors to environments)	Remarked that health promotion and education should not just tell people to eat healthy diets but should also create a healthy environment.	3 gold, 3 green, 1 blue	16
3. Gym, jogging, walking, running	In this, we grouped all behavior that would impact physical activity at the individual level as identified by the stakeholders. Some of the proposed strategies included formulation of regulation that required all new apartments to put up gym facilities in the building, provision of gym at workplaces	3 greens, 2 blues	8
4. School curricula on nutrition and health promotion	This was an existing strategy within the Kenya Comprehensive School Health Policy [19]. It was remarked that the curriculum was comprehensive, but the question was raised as to whether the curricula were being implemented as prescribed. This was identified as a potential question for research.	2 gold, 1 blue	7
5. Integration of Physical Education (PE) into the new Competency-Based Education policy in Kenya [19].	This was identified as a policy-based strategy. It was acknowledged that the PE was incorporated in the former school curriculum as per the Kenya Comprehensive School Health Policy [19]. However, the participants considered this as not having been very effective citing a lack of adequate, safe, and suitable PE facilities particularly for schools in the urban center who have limited space. They also queried whether adequate time was allocated for physical activity in the schools.	1 gold, 1 green, 2 blues	7
6. ‘Control’ of public transportation	The stakeholders discussed this as a policy-based strategy towards the provision of accessible, adequate, and safe infrastructure that would increase the use of public transport in Kenya.	2 gold	6
7. Trail messages: use technology to enhance health promotion messages	This was identified as a strategy that would help modify behavior patterns encouraging increased levels of physical activity and intake of healthy foods. Stakeholders envisioned that an app or text messages via mobile phone would act as reminders or prompts for one to do their daily PA or to check their energy intake. An example of reminders sent through trail messages received from M-PESA [20] mobile money app was given. This was considered futuristic, but it was mentioned that there was already an app being used in the country for the management of hypertension and diabetes [21]. Within this app, there is a function that sends alerts to technicians and patients. If one’s appointment was due, or if one missed their appointment, one receives a reminder. A similar system was proposed for the prevention and control of overweight and obesity in Kenya. Stakeholders identified that such a strategy would act at the behavior modification level.	1 blue, 2 green	5
8. Emphasis on health education in media channels	Stakeholders identified this as a health promotion strategy that would address both the environment and behavior determinants of overweight and obesity. ‘iNooro’ TV and Radio stations [22], the largest vernacular stations in Kenya were given as good examples of local media channels that were already involved in health education.	1 green	2
9. Health promotion in health centres through health talks and display of messages on posters and advertising screens.	The morning health talks given in the health centers were described in detail. These sessions were considered very meaningful and “said a lot”. They also highlighted that this was an intervention at the grass-root level in the counties.	1 green	2
10. Fads, games, and competitions	Stakeholders identified that a number of these are seen to take place as part of nutrition-focused interventions. They noted that some are very popular but without specific medical grounding. Some scientists had raised complaints about such programs. These were considered to impact individual behavior patterns.	1 green	2
11. Social support networks	These networks were seen to spring from some of the fads or other interventions. The example given was the aggressive social media support groups for quail consumption witnessed in Kenya between the years 2013 and 2014 [23, 24]. These support networks were considered effective if the health-promoting product or behavior being supported by the group was scientifically sound. These would impact on sociocultural environments and behavior patterns.	2 blue	2
12. Launching a healthy foods guideline as a national strategy; for example, through the Institute of Food and Agricultural Sciences	This was identified as a policy-based strategy.	2 blues	2
13. Media-based health promotion program	An example given for this was the Slim Possible media program [25] that was featured in Kenya a few years back by the CITIZEN TV media house [22].		0
14. Health promotion strategies that increase the uptake of physical activity			0
15. Introduction of fat tax	It was noted that this discussion was well underway but still at the discussion stage in Kenya [26]. This was considered a policy-based intervention.		0
16. Creation and use of patriotic songs in health promotion	A famous patriotic song that was considered to have encouraged citizens to engage in farming activities was given as an example. Stakeholders present jointly sang along the famous Swahili line ‘*wakulima ongezeni kilimo’.*		0
17. The establishment of a health promotion department at the Ministry of Health and health promotion chapters within their Country health departments			0
18. School feeding programs [19]	Stakeholders remarked that these programs had been in operation for a while now. They commented that the programs were considered fairly effective.		0
19. Health promotion within the antenatal care settings	Commented that antenatal care centers offered a lot of teachings and monitoring to the perinatal and up to five years of baby’s life. Stakeholders discussed that very many interventions are offered in these clinics. A special mention here was the ANC booklets offered to mothers.		0
20. Promotion of agriculture and production of high fibre foods	Stakeholders discussed that Kenya is an agricultural nation. The country produced a lot of good crops, but this has not been looked at as a strategy for health promotion. Stakeholders said that this can be enhanced to inform or positively impact the food environment in the country.		0
21. Have charges for air travel computed per body weight	This strategy raised a lot of debate on human rights and ethics. Discussion around who would receive the income from these charges took place with stakeholders debating whether the proceeds would go to the airline companies or the government as taxes. This way, people would be motivated to lose weight to pay less for travel. This strategy was not deleted from the list but after discussion, the stakeholders considered it not feasible and somewhat unethical.		0
22. Promotion of healthy cooking methods	The use of air fryer was given as an example. On behalf of one of the sub-groups, a stakeholder explained that an air fryer works by circulating hot air around the food and that it was considered a healthy alternative to deep-fried groups.		0
23. Promotion of specifically identified diets such as the Mediterranean diet [27]	A remark was made that the scientific data on the composition of these diets and their effect on health would need to be sourced for the consideration of such a strategy.		0
24. Strategies that address diet and nutrition at the family level not only individual level	It was observed that most of what people eat is influenced by the home setting. An example given was that what children ate was influenced by their parents or households where they lived. So, to fully determine the children’s diet, one would need to know what is being consumed in the homes. This also brought about the issue of food affordability.		0

^a^Each participant was given 3 colored stickers; gold, green, and blue. Each was asked to stick the golden sticker against the strategy that one ranked as number 1 (highest ranking). The green stickers against the strategy that one considered as number 2 and the blue sticker for the strategy that one ranked as number 3

^b^Gold sticker awarded a weighted score of 3, a green sticker awarded a weighted score of 2, and the blue sticker awarded a weighted score of 1

#### Outcomes from meetings held after the workshop

At the joint review meeting held right after the workshop, the four stakeholders present noted that participants had remained fully engaged in the discussions. We reflected on the feedback comments given at the workshop where the participants had expressed their willingness to remain engaged in the rest of the research. The participants had remarked that it was great to have been involved at the very early stage of research. Many reported that in other studies, they would often be engaged in the late stages during the dissemination of research findings. It was however recommended that more stakeholders be involved in the facilitation roles in future workshops. It was noted that holding the workshop on a Friday may have limited attendance for the targeted population. In the future, the engagement sessions would be held in the middle of the week, with a very early morning start time. The field researcher was tasked to set up the joint communication platform that the stakeholders had proposed during the workshop. During this meeting, the field researcher and one stakeholder dispensed transport tokens to the workshop participants through the mobile money platform (M-PESA). Each stakeholder received a token of Kshs. 2000. The award of token was decided upon during the field research and was not part of the original research plan.

In the 2nd meeting held after the workshop, a plan for the report writing was prepared. Tentative publications of the workshop deliberations were discussed in detail. A plan for the data transcription process was prepared.

### Intermediate and long-term outcomes

We are still in the early phase of our research and engagement process. We are therefore not able to assess and report intermediate and long-term outcomes.

As a follow up communication to the stakeholders, a thank you email and electronic thank you card was sent to all who had accepted to support the research, including those absent from the workshop. A second email was sent a few weeks later to share all the slide presentations used in the workshop and photos taken during the workshop. Finally, all participants who attended the workshop were awarded a certificate of participation by Griffith University. These were dispatched on email with hard copies stored for delivery later.

## Discussion

The findings of this study provide us with a context specific, empirical foundation for identification and selection of potential strategies for the prevention and control of overweight and obesity in Kenya, as proposed by stakeholders. The stakeholders proposed high level, broad strategies, and scenarios that they would like the research team to investigate. In the next stage of this study, our research will help define what the proposed broad scenarios look like in practical terms, investigate the effects of the specific actions on health and assess cost-effectiveness.

The strategies proposed by the stakeholders align with the current prevention and control strategies for overweight and obesity in Kenya [19, 28, 29]. The highest-ranked strategies were: a research-based strategy for the identification of the nutritional value of indigenous foods, health promotion strategies that focus on not only education but also creation of healthy environments, physical activity behavior such as gym attendance, jogging, walking, running at the individual level, implementation of school curricula on nutrition and health promotion and integration of physical education into the new Competency-Based Education policy in Kenya, and, control of public transportation as a policy-based strategy that would increase the use of public transport in Kenya. The stakeholders’ propositions align well with some of the WHO ‘best buys’ and recommended interventions for NCD control. These include: the implementation of nutrition education and counselling in school settings to increase the intake of fruits and vegetables, the whole of school programs that support physical activity, mass promotion of intake of fruits and vegetables, implementation of community-wide public education and awareness on uptake of physical activity, and inclusion of health promotion as part of the routine primary health care services [9, 30].

Compared against the categories of obesity determinants and solutions as outlined by Swinburn, Gilland Kumanyika [8] most of the strategies proposed by the stakeholders were seen to focus on the environments and behaviors implementation levels. These are strategies that focus on change at the environmental level by targeting various systemic and environmental drivers of the obesity burden such as policy and economic systems that enable and promote high growth and consumption, and food supply and marketing environments that promote high energy intake. The strategies that focus on change at the behavior level target high food and energy consumption patterns with associated low physical activity levels [8]. Notably, the stakeholders did not propose any strategy for intervention at the physiological level such as drugs and surgery. This may be because the stakeholders may not have perceived the obesity burden in the country to be at a stage where surgery and drugs were required. Alternatively, the stakeholders may have considered these interventions as expensive and not feasible in Kenya, or they may have considered surgery and drugs as ineffective strategies for the control and prevention of overweight and obesity. On the other hand, with an understanding of the potentially huge gains associated with preventive health strategies, the stakeholders may have opted to focus on prevention as opposed to control strategies at the treatment level.

In Kenya, stakeholder engagement for health is identified as part of the principles and approaches that guide the current national strategy for the prevention and control of NCDs [29]. Worth noting is that published literature on stakeholder engaged research in health is limited and what is available is largely focused on high income countries. Ward, Vaughn and, Story [31] conducted a stakeholder meeting in the United States to select top priorities for obesity prevention research in early care and education settings. The researchers used a conference format where experts first spoke to the stakeholders and thereafter roundtable discussions were held to identify research gaps. A list of priorities was compiled and emailed to all stakeholders along with an anonymous online survey for them to choose the three to five recommendations that they felt were the “highest priority” and to rank each one selected on importance (high to low). The research team created a weighted score and identified 24 priority research areas. The group of 43 stakeholders in this study was made up of research experts, leaders from national health agencies and early care and education professionals. The choice of which groups of stakeholders to include in a study is usually informed by the research question under investigation [13].

Another study was the 2017 stakeholder engagement project that was conducted by Lindson, Richards-Doran, Heath and, Hartmann-Boyce, on behalf of the Cochrane Tobaco Addiction Group (TAG) [32]. The aim of the project was to identify areas where further reasearch was needed in the areas of tobacco control and smoking cessation, by involving Cochrane TAG’s stakeholders. As a whole, the project included two surveys and one stakeholder workshop. The included stakeholders were deemed to have an interest in tobacco smoking. The team used purposive sampling to ensure that partcipants represented a range of stakeholder groups and organisations. At the workshop, the participating 43 stakeholders held roundtable discussions to identify top priority research categories from the top 10 identified in the survey. At the end of the workshop, each individual was asked to vote their top three research categories that they thought should be prioritised in future research. They used coloured dots on cards for this exercise to derive a score for each research category. A final axample is the Aidem [33] qualitative study that described the views of 27 stakeholders on criteria and processes for (health) priority setting in Norway. The purposively selected sample of stakeholders expressed their views through semi-structured interviews and focus groups. This too was conducted in a high-income country. The stakeholders included extended beyond the policy makers to include hospital administrators, practitioners, university students and seniors. These studies provide evidence regarding similar stakeholder engagements in priority setting within various areas of health. This supports the argument that the involvement of stakeholders is considered key to successful priority setting for NCD prevention and control [29, 34].

## Limitations and strengths of the study

Purposive sampling has inherent selection bias hence generalizability of the results is limited. Despite this limitation, the findings may be relevant in other low- and middle-income countries with similar setting as Kenya.

The selection of participants was limited to stakeholders involved in making decisions for health in Kenya at the national policy level. However, by incorporating stakeholders from several civil society organizations, we considered that the public would be effectively represented. Due to time and funding constraints on the project, the stakeholder recruitment was done within a limited timeframe of 1 month. Further, due to the nature of work for the recruited stakeholders, work commitments made it difficult for some of them to attend our workshop. Nevertheless, we did meet our target number of attendees and we achieved great representation from multiple sectors involved in priority setting for NCD control in health.

The audio recording done in the workshop was captured at a low volume and had background room noise. This presented a challenge in the transcription process. To ensure that all conversations were transcribed, the transcription was reviewed by 3 people, two of whom are authors of this paper (MNW and LKB). Though the discussions within the smaller groups were not audio-recorded, we do not consider this to have interfered with the accurate recording of the discussions that took place. We utilized workshop notes from the sub-group scribes. These were on flipchart recordings for one sub-group and in a PowerPoint presentation for another sub-group. A report of the workshop has been shared with all stakeholders before the publication of this manuscript. A key strength of our study was the engagement of a wide range of stakeholders at a very early stage of our research. This has improved the quality and scope of our research and will assist in the promulgation of the findings, accelerating the adoption of our findings into practice.

## Conclusion

The stakeholders identified and ranked strategies for the prevention and control of overweight and obesity in Kenya.

The broad strategies identified here could inform policymakers and other stakeholders who may be seeking to identify context-specific strategies for prevention and control of overweight and obesity. The findings inform future overweight and obesity prevention research and policy in Kenya and similar settings.

## Supplementary Information

**Additional file 1:Supplementary file 1.** Displayed lists of all the identified strategies at the workshop. These was the list of identified strategies that was compiled and displayed at the front of the workshop room. The stakeholders’ ranking is also displayed through the coloured stickers they have put against specific strategies for the.

**Additional file 2:Supplementary file 2.** Day’s Program. This is a copy of the program followed for the one day stakeholder workshop held. This paper reports the results from the Workshop activity II that was held in the afternoon session within the one day stakeholders workshop.

## Data Availability

All data generated or analysed during this study are included in this published article and its supplementary information file 1  and 2.

## Abbreviations

ACE: Assessing cost-effectiveness
BMI: Body Mass Index
DALYs: Disability-adjusted life years
KALRO: Kenya Agricultural & Livestock Research Organization
KEMRI: Kenya Medical Research Institute
MoH: Ministry of Health
NCDs: Non-communicable diseases
PE: Physical education
SRQR: Standards for reporting qualitative research
TAG: Tobaco Addiction Group
