# Supplementary material for: Stakeholder-engaged research: strategies for the prevention and control of overweight and obesity in Kenya
Source: BMC Public Health. 2021 Sep 6;21:1622. doi: 10.1186/s12889-021-11649-0 (PMC8420014; doi:10.1186/s12889-021-11649-0)

**Title**

**Stakeholder-engaged research: Strategies for the prevention and control of overweight and obesity in Kenya**

**Corresponding author**

**Mary Njeri Wanjau**

School of Nursing Sciences, University of Nairobi

School of Medicine, Griffith University, QLD 4222, Australia.

Gold Coast campus, Parklands Drive, Southport, QLD, 4222

[mary.wanjau@griffithuni.edu.au](about:blank)

+61 (0) 484274134

**Co-authors**

**Dr. Lucy Kivuti-Bitok**

School of Nursing Sciences, University of Nairobi

P.O. Box 19676-00200, Nairobi, Kenya

[lukibitok@uonbi.ac.ke](about:blank)

**Dr. Leopold N. Aminde**

Non-communicable Disease Unit, Clinical Research Education, Networking & Consultancy, Douala, Cameroon

School of Medicine, Griffith University, QLD 4222, Australia.

Gold Coast campus, Parklands Drive, Southport, QLD, 4222

[l.aminde@griffith.edu.au](about:blank)

**Prof. Lennert Veerman**

School of Medicine, Griffith University, QLD 4222, Australia.

Gold Coast campus, Parklands Drive, Southport, QLD, 4222

[l.veerman@griffith.edu.au](about:blank)

**Displayed lists of all the identified strategies and ranking done at the workshop**


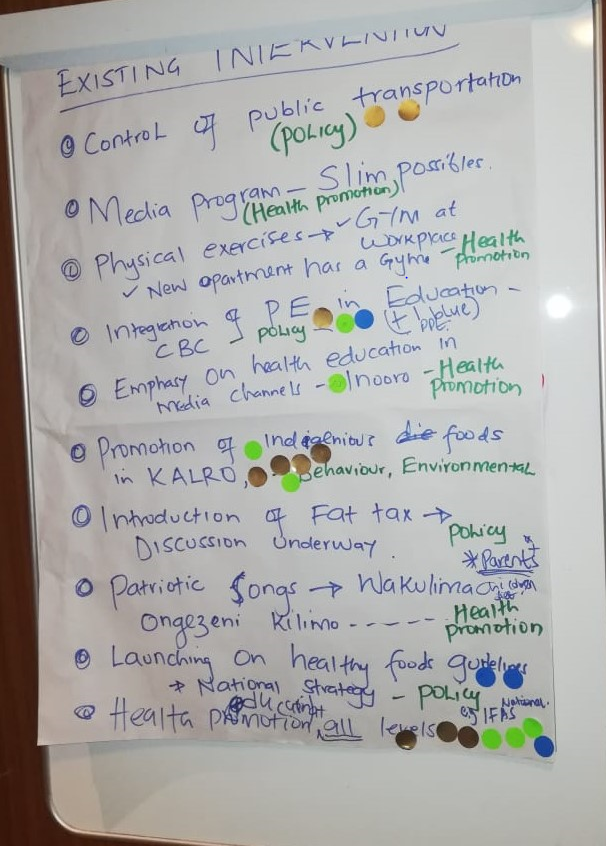


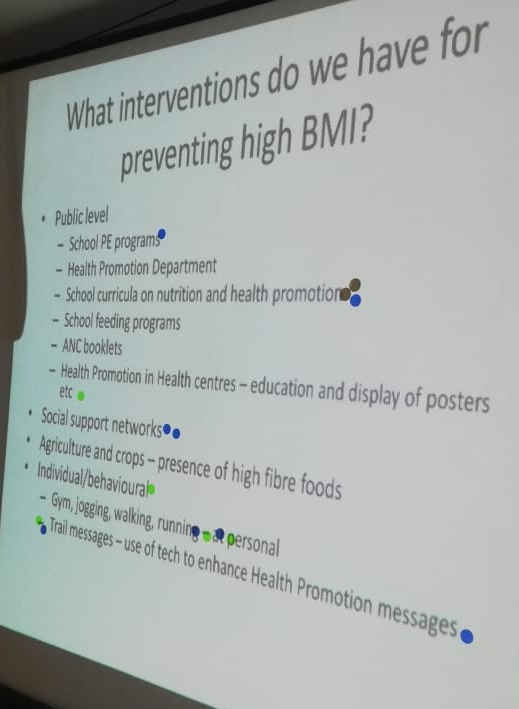

Supplement: Supplementary file 1 — Additional file 1:Supplementary file 1. Displayed lists of all the identified strategies at the workshop. These was the list of identified strategies that was compiled and displayed at the front of the workshop room. The stakeholders’ ranking is also displayed through the coloured stickers they have put against specific strategies for the. [file 12889_2021_11649_MOESM1_ESM.docx]
