# Supplementary material for: Stakeholder-engaged research: strategies for the prevention and control of overweight and obesity in Kenya
Source: BMC Public Health. 2021 Sep 6;21:1622. doi: 10.1186/s12889-021-11649-0 (PMC8420014; doi:10.1186/s12889-021-11649-0)

**Title**

**Stakeholder-engaged research: Strategies for the prevention and control of overweight and obesity in Kenya**

**Corresponding author**

**Mary Njeri Wanjau**

School of Nursing Sciences, University of Nairobi

School of Medicine, Griffith University, QLD 4222, Australia.

Gold Coast campus, Parklands Drive, Southport, QLD, 4222

[mary.wanjau@griffithuni.edu.au](about:blank)

+61 (0) 484274134

**Co-authors**

**Dr. Lucy Kivuti-Bitok**

School of Nursing Sciences, University of Nairobi

P.O. Box 19676-00200, Nairobi, Kenya

[lukibitok@uonbi.ac.ke](about:blank)

**Dr. Leopold N. Aminde**

Non-communicable Disease Unit, Clinical Research Education, Networking & Consultancy, Douala, Cameroon

School of Medicine, Griffith University, QLD 4222, Australia.

Gold Coast campus, Parklands Drive, Southport, QLD, 4222

[l.aminde@griffith.edu.au](about:blank)

**Prof. Lennert Veerman**

School of Medicine, Griffith University, QLD 4222, Australia.

Gold Coast campus, Parklands Drive, Southport, QLD, 4222

[l.veerman@griffith.edu.au](about:blank)

**The Days’ program**


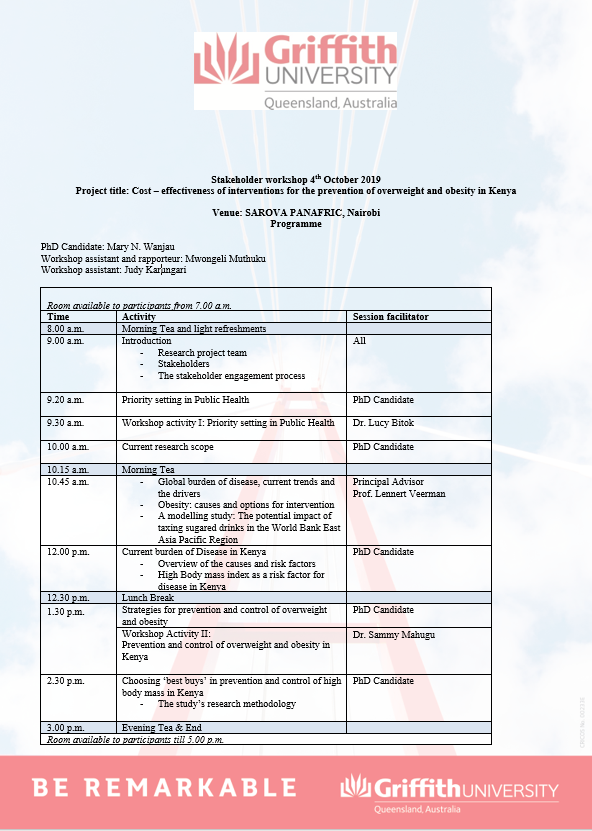

Supplement: Supplementary file 2 — Additional file 2:Supplementary file 2. Day’s Program. This is a copy of the program followed for the one day stakeholder workshop held. This paper reports the results from the Workshop activity II that was held in the afternoon session within the one day stakeholders workshop. [file 12889_2021_11649_MOESM2_ESM.docx]
